# Population genetics of an alien whitefly in China: implications for its dispersal and invasion success

**DOI:** 10.1038/s41598-017-02433-5

**Published:** 2017-05-22

**Authors:** Hong-Ran Li, Hui-Peng Pan, Yun-Li Tao, You-Jun Zhang, Dong Chu

**Affiliations:** 1 Key Lab of Integrated Crop Pest Management of Shandong Province, College of Agronomy and Plant Protection, Qingdao Agricultural University, Qingdao, 266109 P. R. China; 2 Department of Entomology, South China Agricultural University, Key Laboratory of Bio-Pesticide Innovation and Application of Guangdong Province, Guangzhou, 510642 P. R. China; 3 Institute of Vegetables and Flowers, Chinese Academy of Agricultural Sciences, Beijing, 100081 P. R. China

## Abstract

Invasive genotypes may be associated with their ability to access the invasion habitat. The whitefly, *Bemisia tabaci* Q, has been an important agricultural pest in China since 2008. In order to identify the invasion routes and to provide insight into its invasion success in China, we analyzed the composition, distribution, and genetic diversity of mitochondrial haplotypes of *B*. *tabaci* Q. Samples were obtained from 23 provincial level administrative units in 2011, and analyses conducted based on the *mtCOI*. Our results revealed five haplotypes (abbreviated as Q1H1-Q1H5) were present in the Q1 subclade based on 773-bp *mtCOI* fragment analysis. The diversity of haplotypes indicated the *B*. *tabaci* Q populations were derived from multiple invasion sources originating from the western Mediterranean region. Among the haplotypes, Q1H1 was dominant, followed by Q1H2. The whitefly populations were generally characterized by low levels of genetic diversity based on the 773-bp *mtCOI* fragment. Similar results were obtained when the 657-bp fragment was analyzed using the procedure in a previous report. Potential mechanisms contributing to the dominance of the Q1H1 in China are also discussed. These results will be helpful in revealing the mechanisms that enabled the successful invasion of *B*. *tabaci* Q into the country.

## Introduction

Biological invasions often inflict tremendous damage to agriculture, forestry, and natural ecosystems in the introduced regions^1^. Studies involving the genotype and/or genetic diversity of the invasive species will help in determining the invasion mechanisms involved and possibly provide information that will be useful in the sustainable management of species invasions^2–4^. The invasive genotypes represented by the predominant haplotypes, for example, may be associated with the invasion route and a set of traits that facilitate their ability to access the invasion habitat^5–7^. Alternatively, molecular studies have shown genetic diversity in the invaded regions may contribute to the successful invasion of the alien species^8–10^. The analyses of genotype and genetic diversity of invasive species may be conductive to the selection of appropriate management measures such as the interception of the potential invasive individuals and introduction of their natural enemies.

The whitefly, *Bemisia tabaci* (Gennadius) (Hemiptera: Aleyrodidae), is one of the most destructive plant pests of major great agriculture importance, causing damage to its hosts directly through feeding on plant sap or indirectly through virus transmission^11^. Most species and varieties of the *B*. *tabaci* complex are restricted to specific geographic regions, but several of them, such as the putative species of MEAM1 (hereafter referred to as *B*. *tabaci* B) and MED (hereafter referred to as *B*. *tabaci* Q), are highly invasive and have become distributed worldwide^12–14^. Since 2003, *B*. *tabaci* Q has rapidly spread throughout China in a variety of field crops, displacing the previously established invasive whitefly, *B*. *tabaci* B, which has been the globally dominant species for approximately two decades^15–20^. However, the invasion route(s) of *B*. *tabaci* Q and mechanism underlying its successful invasion into China remain poorly understood.

The mitochondrial cytochrome c oxidase I (*mtCOI*) gene has become a very useful tool in exploring the evolutionary aspects of species invasions ever since it was used to serve as the marker of choice in the context of the DNA barcode system^21^. De Barro and Ahmed^3^ used the *mtCOI* haplotype (657 bp fragment) to identify genetic networks of *B*. *tabaci* Q on a global scale. Eighty-five haplotypes of this pest have been described to date^3^. Three distinct mitochondrial variants of *B*. *tabaci* Q whiteflies identified by McKenzie *et al*.^22^ were widely distributed throughout North America. Nuclear data combined with the *mtCOI* data strongly support the inference of multiple, independent *B*. *tabaci* Q invasions into North America from at least three different introductions^23^. A previous study^24^ revealed that *B*. *tabaci* Q could be differentiated into two subclades based on the *mtCOI* gene sequence. These two subclades have been labeled as *B*. *tabaci* subclades Q1 and Q2. *B*. *tabaci* Q1 mainly distributes in the western Mediterranean region while Q2 mainly in the eastern Mediterranean region. Using *mtCOI* and nuclear (microsatellite) DNA, an investigation of genetic diversity by Chu *et al*.^25^ indicated that *B*. *tabaci* Q in China (Shandong Province) may have originated from the western Mediterranean region. The genotype and genetic diversity of *B*. *tabaci* Q, using the *mtCOI* gene, remain unknown from most regions across China. Identifying these will be helpful in tracing the invasion routes of *B*. *tabaci* Q and in determining the mechanism underlying its successful invasion into China.

The present study involved sampling an extensive geographic area covering the majority of the known *B*. *tabaci* distribution in China during 2011, to identify the genotype and genetic diversity of *B*. *tabaci* Q by analyzing the *mtCOI* gene. The results from these analyses were used to authenticate existing theories on the initial source of the invasion(s) as well as helping to define the likely mechanisms underlying the successful invasion(s) of *B*. *tabaci* Q into China.

## Results

In total, 648 individual whiteflies from 50 different collections were successfully sequenced and analyzed. Of those individuals, 396 (61%) were *B*. *tabaci* Q, 214 (33%) were *B*. *tabaci* B, and 38 (6%) were miscellaneous other “species” of the *B*. *tabaci* species complex (abbreviated as “others”). The other forms were mainly four *B*. *tabaci* “species” indigenous to China, including Asia II 2, Asia II 6, Asia I, and China1^26^.

The haplotypes that were determined refers to Q haplotypes with at least two identical sequences. Among the 396 well-defined *B*. *tabaci* Q individuals (based on the 773 bp fragment), 371 individuals’ haplotypes have been determined: 280 (76%) were the Q1H1 haplotype, 79 (21%) were the Q1H2 haplotype, and 12 (3%) were other haplotypes (classified as Q1H3-Q1H5). The Genbank numbers of haplotypes Q1H1-Q1H5 were as follows: KY468416, KY468417, KY468418, KY468419, and KY468420. Using the 657 bp fragment, as defined by De Barro and Ahmed^3^, 386 individuals’ haplotypes have been determined: 306 (79%) individuals were identified as the Hap1 haplotype (Genbank No. KY468421), and 80 (21%) individuals were the Hap2 haplotype (Genbank No. KY468422).

### “Species” composition and geographical distribution of *Bemisia tabaci*

**Figure 1:**
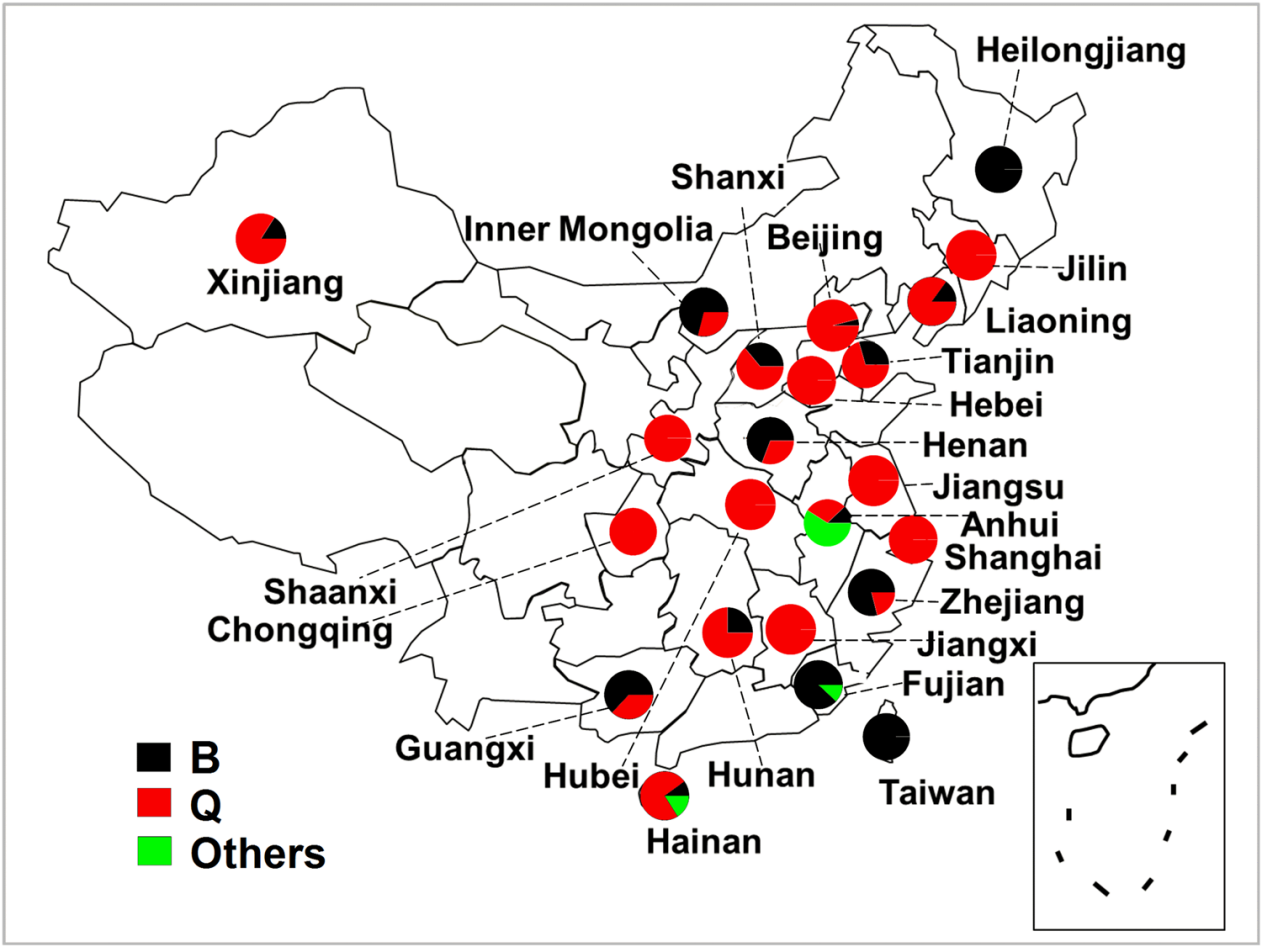
Distribution of *Bemisia tabaci* species in China in 2011. Circles denote proportion of *B*. *tabaci* B (black), Q (red) and other species (green) in collections. This China map was created using Microsoft PowerPoint (version 2010) by author Hui-Peng Pan.

Of the 50 whitefly samples collected from 23 provinces in 2011, 19 of the collections from eight provinces (Jilin, Hebei, Shannxi, Jiangsu, Chongqing, Hubei, Shanghai, and Jiangxi) contained only *B*. *tabaci* Q, whereas three collections from Heilongjiang and Taiwan contained only *B*. *tabaci* B, Other *B*. *tabaci* non-B/Q “species” were found in seven collections from Anhui, Fujian, and Hainan (Table 1). Surprisingly, the percentage of *B*. *tabaci* non-B/Q “species” was >50% only in Anhui Province. Twenty-one collections from the remaining provinces contained mixed amounts of *B*. *tabaci* B and Q. At the provincial level, *B*. *tabaci* Q was not the predominant form (>50% of sampled individuals) in only five provinces out of 13 containing mixed species (Fig. 1). Taken as a whole, the samples from agricultural regions showed a prevalence of *B*. *tabaci* Q throughout much of China.

**Table 1:** Collections sampling localities and details for *Bemisia tabaci* in China in 2011.

Regions	Location	Host	Number of *mtCOI* sequence	Number of Q	Q haplotype Based 657 bp		Q1H1	Q haplotype Based 773 bp		Number of B	Number of other whitefly species
					Hap1	Hap2		Q1H2	Others		
Northwestern China	Tulufan, Xinjiang	Cotton, eggplant	32	27	17	10	17	10	0	5	0
	Yangling, Shaanxi	Tomato	4	4	4	0	3	0	0	0	0
Southwestern China	Chongqing	Tomato, eggplant, cucumber	9	9	4	5	3	5	1	0	0
Northern China	Huhehaote, Inner Mongolia	Tomato	17	5	4	1	4	1	0	12	0
	Yuncheng, Shanxi	Cotton	25	16	13	2	10	2	1	9	0
	Changping, Beijing	Tomato, cotton	28	27	22	5	21	5	1	1	0
	Tianjin	Tomato, cucumber, eggplant	17	10	10	0	6	0	2	7	0
	Shijiangzhuang, Hebei	Tomato, cotton, eggplant	16	16	12	4	9	3	2	0	0
Central China	Wuhan, Hubei	Cotton, eggplant, cucumber	22	22	21	1	19	1	2	0	0
	Changsha, Hunan	Tomato, eggplant	4	3	3	0	3	0	0	1	0
	Luoyang, Henan	Tomato, cotton, eggplant	39	12	10	2	10	2	0	27	0
Northeastern China	Changchun, Jilin	Tomato, cotton, eggplant	24	24	20	2	19	2	0	0	0
	Daqing, Helongjiang	Soybean	15	0	—	—	—	—	—	15	0
	Chaoyang, Liaoning	Tomato	106	88	70	14	64	14	2	18	0
Eastern China	Nanjing, Jiangsu	Tomato, cotton, eggplant	44	44	38	6	38	6	0	0	0
	Hefei, Anhui	Tomato, cotton, eggplant	49	14	10	2	8	2	1	6	29
	Fuzhou, Fujian	Tomato, eggplant	58	0	—	—	—	—	—	52	6
	Fengxian, Shanghai	Tomato, eggplant	13	13	11	2	10	2	0	0	0
	Nanchang, Jiangxi	Eggplant	23	23	22	0	22	0	0	0	0
	Taiwan	Tomato, eggplant	9	0	—	—	—	—	—	9	0
	Hangzhou, Zhejiang	Tomato, cotton, eggplant	19	4	4	0	3	0	0	15	0
Southern China	Nanning, Guangxi	Tomato, cucumber, eggplant	56	21	8	13	8	13	0	35	0
	Haikou, Hainan	Tomato, eggplant	19	14	3	11	3	11	0	2	3
	Total		648	396	306	80	280	79	12	214	38

The haplotypes that were determined refers to haplotypes belonging to Q where there were at least two identical sequences.

### Haplotype composition and geographical distribution of *Bemisia tabaci* Q

**Figure 2:**
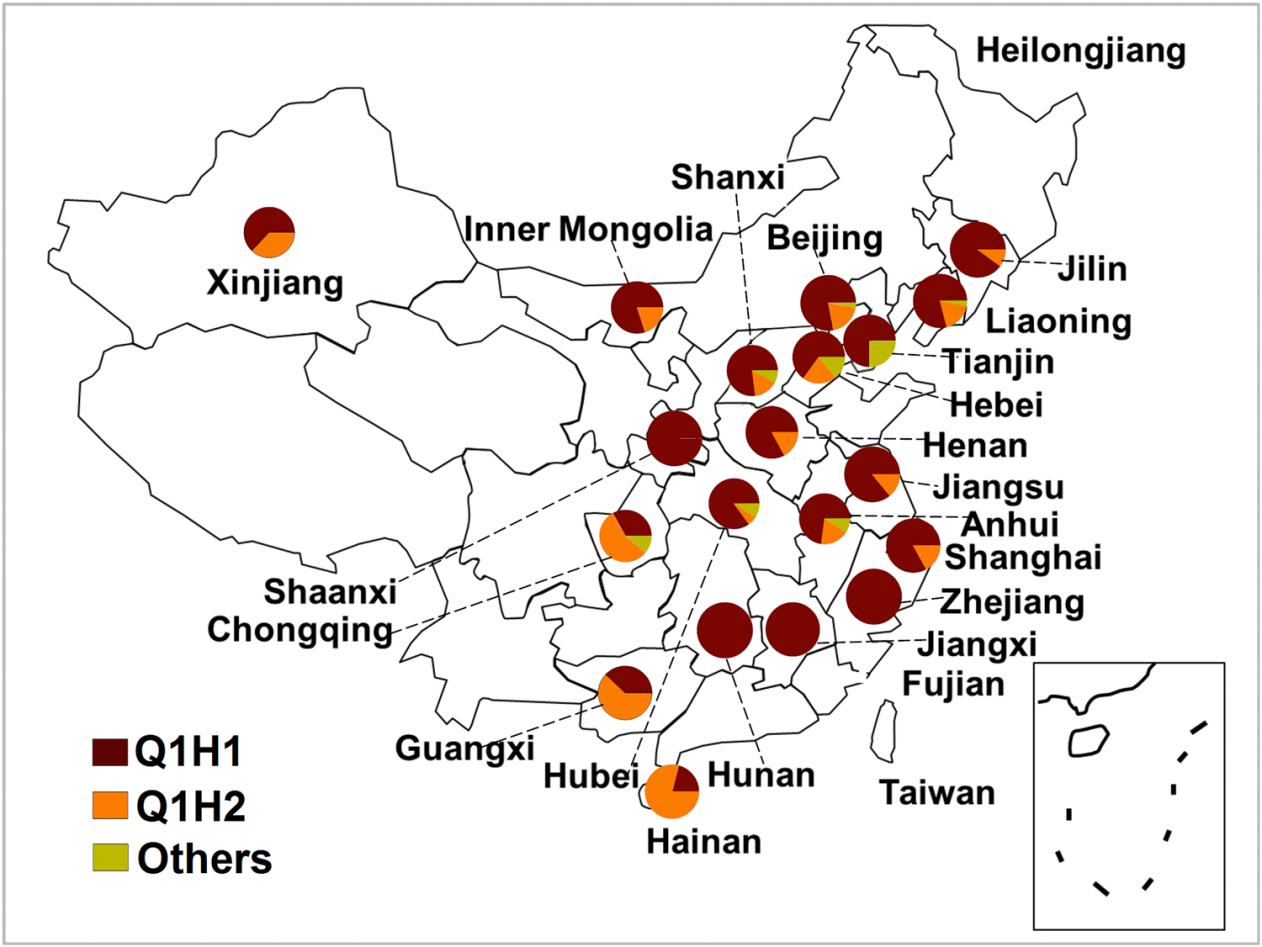
Distribution of haplotypes among Bemisia tabaci Q in China in 2011 based on 773-bp mtCOI fragment. Circles denote proportion of Q1H1 (brown), Q1H2 (yellow) and other haplotypes (light green) in collections. This China map was created using Microsoft PowerPoint (version 2010) by author Hui-Peng Pan.

Based on the 773 bp length fragment, a total of six haplotypes, designated as Q1H1-Q1H5, were identified (Fig. 2). Other than the two common haplotypes, Q1H1 and Q1H2, the four other less frequently encountered haplotypes were apparently restricted to eight provinces as follows: 1) Q1H3 was distributed in Tianjin, Hebei, and Anhui; 2) Q1H4 was widespread in Beijing, Shanxi, Hubei, and Liaoning; 3) Q1H5 was distributed in Hebei and Tianjin.

Based on the 657 bp length fragment, as defined by De Barro and Ahmed^3^, only two haplotypes, Hap1 and Hap2, were identified (Supplementary Fig. S1). Of all collections containing at least one individual of *B*. *tabaci* Q, only Hap1 individuals occurred in Jiangxi, Zhejiang, Shannxi, Tianjin, and Hunan provinces, whereas Hap2 individuals were not found in any of these provinces. Hap1was dominant (>50%) in each of the remaining mixed-haplotypes provinces except Hainan, Guangxi, and Chongqing.

### Mitochondrial COI diversity and genetic differentiation of *Bemisia tabaci* Q

Genetic diversity analysis based on the *mtCOI* gene varied among different geographical populations. Based on the 773 bp fragment, the haplotype diversity (*Hd*) values found in different provinces ranged from 0.000 to 0.639 with an average *Hd* value of 0.372 (Table 2). Based on the 657 bp fragment as defined by De Barro and Ahmed^3^, the *Hd* of different provinces ranged from 0.000 to 0.556 with an average *Hd* value of 0.290 (Supplementary Table S1). On the other hand, Fu’s *F* and Tajima’s *D* indices, based on 773 bp and 657 bp fragment, showed no significant difference between different provinces.

**Table 2:** Genetic diversity indices of *Bemisia tabaci* Q collections in China in 2011 based on 773-bp *mtCOI* fragment.

Location* (Number of individuals tested)	*S*	*η*	*H*	*Hd* (SD)	*π* (SD)	*π* (JC)	*K*	*D* (*p*)	*Fs* (*p*)
Xinjiang (27)	1	1	2	0.484 (0.054)	0.00063 (0.00007)	0.00063	0.484	1.39947 (*ns*)	1.514 (ns)
Chongqing (9)	2	2	3	0.639 (0.126)	0.00101 (0.00026)	0.00101	0.778	0.19590 (*ns*)	−0.108 (*ns*)
Inner Mongolia (5)	1	1	2	0.400 (0.237)	0.00052 (0.00031)	0.00052	0.400	−0.81650 (*ns*)	0.090 (*ns*)
Shanxi (13)	2	2	3	0.410 (0.154)	0.00056 (0.00023)	0.00056	0.436	−0.90920 (*ns*)	−0.790 (*ns*)
Hebei (14)	3	3	4	0.571 (0.132)	0.00084 (0.00024)	0.00084	0.648	−0.95732 (*ns*)	−1.362 (*ns*)
Beijing (27)	2	2	3	0.373 (0.101)	0.00050 (0.00014)	0.00050	0.387	−0.53597 (*ns*)	−0.490 (*ns*)
Tianjin (8)	2	2	3	0.464 (0.200)	0.00065 (0.00031)	0.00065	0.500	−1.31009 (*ns*)	−0.999 (*ns*)
Hubei (22)	2	2	3	0.255 (0.116)	0.00034 (0.00016)	0.00034	0.264	−1.17515 (*ns*)	−1.310 (*ns*)
Henan (12)	1	1	2	0.303 (0.147)	0.00039 (0.00019)	0.00039	0.303	−0.19492 (*ns*)	0.297 (*ns*)
Jilin (21)	1	1	2	0.181 (0.104)	0.00023 (0.00014)	0.00023	0.181	−0.61772 (*ns*)	−0.137 (*ns*)
Liaoning (82)	2	2	3	0.333 (0.058)	0.00044 (0.00008)	0.00044	0.342	−0.24678 (*ns*)	−0.152 (*ns*)
Jiangsu (44)	1	1	2	0.241 (0.076)	0.00031 (0.00010)	0.00031	0.241	0.06730 (*ns*)	0.551 (*ns*)
Anhui (11)	2	2	3	0.473 (0.162)	0.00066 (0.00025)	0.00066	0.509	−0.77815 (*ns*)	−0.659 (*ns*)
Shanghai (12)	1	1	2	0.303 (0.147)	0.00039 (0.00019)	0.00039	0.303	−0.19492 (*ns*)	0.297 (*ns*)
Jiangxi (22)	0	0	1	0.000 (0.000)	0.00000 (0.00000)	0.00000	0.000	—	—
Guangxi (21)	1	1	2	0.495 (0.060)	0.00064 (0.00009)	0.00064	0.495	1.38372 (*ns*)	1.403 (*ns*)
Hainan (14)	1	1	2	0.363 (0.130)	0.00047 (0.00017)	0.00047	0.363	0.32440 (*ns*)	0.643 (*ns*)

*The indices for the Shannxi, Hunan and Zhejiang samples were not calculated because the numbers of individuals were below 5; *S*, number of polymorphic (segregating) sites; *η*, total number of mutations; *H*, number of haplotypes; *Hd*, haplotype diversity; *π*, nucleotide diversity; *K*, average number of nucleotide differences; *π*(JC), nucleotide diversity with Jukes and Cantor correction; *D*, Tajima’s *D* statistic; *Fs*, Fu’s *F* test statistic; ns, not significant.

### Mitochondrial COI haplotype network of *Bemisia tabaci* Q

**Figure 3:**
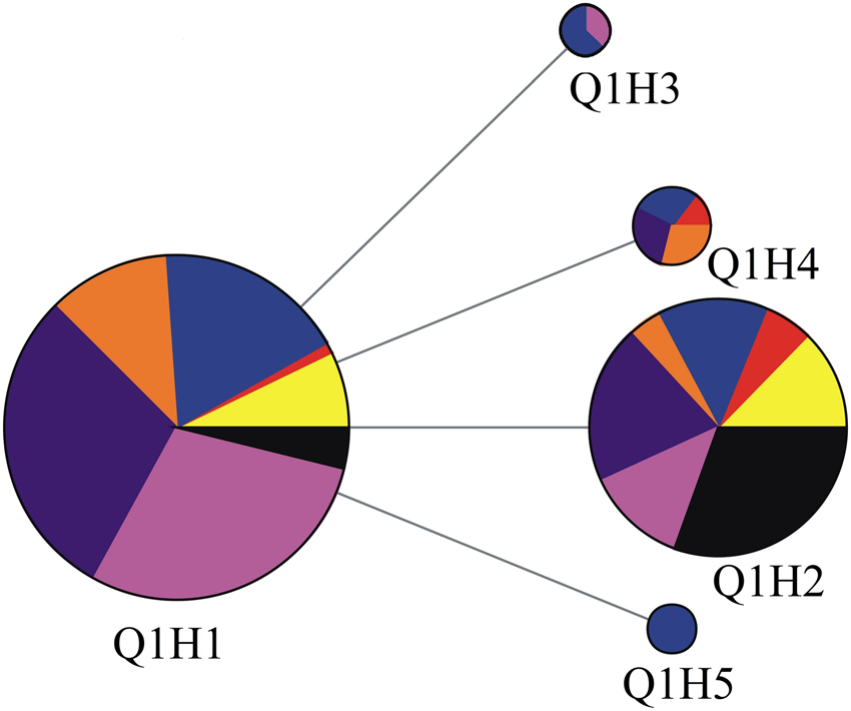
Network analyses of haplotype relationships based on 773-bp *mtCOI* fragment. Colors within the nodes: red, Southeastern China; orange, Central China; yellow, Northwestern China; purple, Northeastern China; blue, Northern China; pink, Eastern China; black, Southern China.

Based on the 773 bp fragment, two major haplotypes were formed in the haplotype network tree, which displayed a distinct star-like pattern with the most common haplotypes in the star’s center. A total of six *mtCOI* haplotypes were identified in this study, among which, Q1H1 and Q1H2 haplotypes were shared by different regions (Table 1) (Fig. 3). The other four haplotypes were mutated from haplotype Q1H1 with their distribution being restricted to eight provinces. Additionally, the haplotype network tree of *mtCOI* only formed two haplotypes (Hap1 and Hap2) which were shared by all sampling regions based on the 657-bp length fragment as defined by De Barro and Ahmed^3^ (Supplementary Fig. S2).

## Discussion

In this study, we obtained *B*. *tabaci* collections from sites covering the majority of the known *B*. *tabaci* distribution in China. This is the first extensive field survey in which the mitochondrial haplotypes and genetic diversity of *B*. *tabaci* Q were investigated in order to determine the initial source, the subsequent spread pattern, and potential invasion mechanism of this pest in China.

The present study demonstrated that the *B*. *tabaci* biotypes, B and Q, are prevalent in most regions of China. Furthermore, *B*. *tabaci* Q has become the dominant “species” throughout the country, which substantiates our previous study showing that the displacement of other *B*. *tabaci* species by *B*. *tabaci* Q has occurred in many regions of the country^15, 19^. However, there are still limited numbers of non-B/Q individuals of indigenous biotypes such as Asia II 2, Asia II 6, Asia I, and China 1 present in some areas in the southern and eastern parts of China^26^.

This study revealed that *B*.*tabaci* Q in China is composed of several haloptypes, including Hap1 and Hap2 (based on the 657 bp gene fragment) or Q1H1-Q1H5 (based on the 773 bp gene fragment). The haplotype composition of the *mtCOI* gene can be very informative in affording clues to the likely origin of invading organisms. Previous studies^27, 28^ involving analysis of the *mtCOI* gene showed that the *B*. *tabaci* Q1 subclade may be distributed primarily in the western Mediterranean countries (e.g. Morocco and Spain) and the Q2 subclade, in the eastern Mediterranean countries (e.g. Israel). Moreover, another previous study demonstrated that all of the *B*. *tabaci* Q in China were grouped into subclade Q1^24^. De Barro and Ahmed^3^ determined that the 657-bp Hap1 (i.e., Q1H1 in this study) and Hap2 (i.e., Q1H2 in this study) haplotypes originated in the western Mediterranean regions. The phylogenetic analysis of NJ trees based on *mtCOI* sequences of ~650-bp revealed that all haplotypes and the sequences that were found only a single time (Genbank No. KY468408- KY468415) clustered to subclade Q1 (Supplementary Fig. S3).Thus, the composition of Q, including Hap1 and Hap2 (657-bp gene fragment) and the Q1H1-Q1H5 haplotypes (773-bp gene fragment), in this study indicates the initial *B*. *tabaci* Q populations originated from western Mediterranean regions. These results are consistent with our previous study that the *mtCOI* haplotypes found in Shandong Province, China may have originated from western Mediterranean countries (such as Morocco and Spain)^25^. Chu *et al*.^25^ identified four distinct haplotypes (haplotype1-haplotype 4, respectively). Among these, their haplotype 1 is analogous to Hap 1 (based on 657 bp) or Q1H1 (based on 773 bp), and haplotype 2 analogous to Hap 2 (based on 657 bp) or Q1H2 (based on 773 bp), while their haplotypes 3 and 4 (belonging to the Q1 subclade) were not found in the present study. In addition, our previous study found that the introduced populations of *B*. *tabaci* Q in China have high spatial genetic heterogeneity possibly resulting from multiple introductions, rapid evolution following one or few introductions, or some combination of multiple introductions and rapid evolution^29^. Thus, the introduced haplotype as identified based on the 773-bp *mtCOI* gene, indicates the Q populations in China may have been introduced from multiple invasion sources.

Our results demonstrated that most of the genetic diversity found in our samples is similar except in individuals from Hubei, Jilin, Jiangsu, and Jiangxi provinces, where it is relatively low. The results suggest that the gene flow among most sampled areas across China is extensive, which is consistent with results published by Chu *et al*.^30^ stating that high gene flow occurred between *B*. *tabaci* Q populations in Shandong Province, China. Previous studies have suggested that genetic diversity is often associated with the ecological adaptation of a species^31–33^. Multiple introductions populations with a distinctive genetic composition, may contribute to the founding population and that the accumulation of diversity can act to facilitate the process of invasion^8, 29, 34^. However, the genetic paradox in species invasions which often experience bottleneck or founder events showed that many invasive species have a low genetic diversity^35, 36^. Chu *et al*.^25^ indicated the loss in haplotype diversity of mitochondrial DNA does not necessarily mean that nuclear allelic diversity should also decline. Our study suggests that the genetic diversity based on mitochondrial DNA is not closely associated with the successful invasion of *B*.*tabaci* Q in China. Thus, we postulate that the nuclear diversity of Q in China might not be lower compared to their source or original population as indicated by Chu *et al*.^25^.

Surprisingly, we found that the Q1H1 is the dominant haplotype in most agricultural regions in China. The invasive genotypes represented by the predominant *mtCOI* haplotypes may be associated with a set of traits such as adaptability to adverse environments, host plant or due to some extent to other external factors, e.g. insecticide applications, that facilitate their ability to successfully colonize the invasion habitat. For *B*. *tabaci* Q, we believe insecticide application may have contributed to the prevalence of Q1H1 in China. Pan *et al*.^20^ demonstrated that *B*. *tabaci* Q was more tolerant of insecticides, and that the overuse of insecticides reversed the *B*. *tabaci* B and Q competitive hierarchy and allowed *B*. *tabaci* Q to displace B in managed landscapes in China. Notably, field populations of *B*. *tabaci* Q have developed high levels of resistance to imidacloprid and thiamethoxam, and the haplotype of *B*. *tabaci* Q was further identified to be the haplotype Q1H1^20, 37, 38^. This phenomenon may likely be a major reason why haplotype Q1H1 is now the predominant haplotype in most agricultural regions in China.

High temperatures may be another factor in explaining the distribution and composition of *B*. *tabaci* “species”^39^. The present study demonstrated that haplotype Q1H2 is especially widespread in southern China where temperatures, rainfall and humidity are considerably higher than in other parts of the country. Another factor that may be contributing to the prevalence of Q1H2 is the effect of inherited endosymbionts, which are common in many arthropods and are known to have major effects on the biology of their hosts. Our recent study showed that, compared to the *Cardinium*-uninfected *B*. *tabaci* Q, *Cardinium*-infected *B*. *tabaci* Q was less competitive. This would explain why *Cardinium*-uninfected *B*. *tabaci* Q have become the dominate form found in field populations in China^40, 41^. It has been suggested that endosymbionts may have played a role in the *B*. *tabaci* Q invasions in southern Italy, acting as a sex-ratio manipulator and possibly benefiting the host fitness^42^. The haplotype-endosymbiont associations of *B*. *tabaci* Q, as well as other biological differences between Q1H1 and Q1H2 should be further explored to better understand the reasons behind the invasion success of *B*. *tabaci* Q in China. Identifying the precise factors may provide the basis contributing to ultimately controlling this harmful pest with appropriate strategies.

## Materials and Methods

### Whitefly collections

Specimens were collected from greenhouses and cultivated fields of cotton, vegetables, and soybeans from 23 provincial level administrative units, covering the majority of the known distribution of *B*. *tabaci* in China during 2011. Collection details, host plants, and number of whiteflies are summarized in Table 1. The majority of the whitefly samples were provided through cooperation with several universities, the Academy of Agricultural Sciences, and local agricultural agencies. Each collection was obtained by randomly sampling using a hand-held aspirator. The whiteflies were then transferred to plastic tubes containing 95% ethanol and preserved at −20 °C until processing. A minimum of four individuals per collection was used to determine the species composition.

### DNA extraction

DNA was extracted from individual whiteflies by placing a single whitefly into a plastic tube, adding 10 μl of DNA lysis buffer, and grinding with a sealed pipet. Extracts were first incubated at 65 °C for 15 min and then 95 °C for 10 min prior to homogenizing in a final volume of 50 μl^43^. The individual whitefly DNA extracts were stored at −20 °C and used for both species and haplotype determinations.

### Determination of *B*. *tabaci* species and *B*. *tabaci* Q haplotypes

Specimens were identified based on the mitochondrial cytochrome oxydase I (*mtCOI*) gene, using the primer pair Cl-J-2195 (5′-TTBATTTTTTGGTCATCCAGAAGT-3′) and L2-N-3014 (5′-TCCAATGCACTAATCTGCCATATTA-3′) as described by Frohlich *et al*.^42, 43^. All PCR reactions were performed in 13 μl solutions containing 1 × buffer, 0.16 mM of each dNTP, 0.5 mM of each primer, 0.5 unit of Taq DNA polymerase, and 2 μl of template DNA. Cycling conditions consisted of an initial denaturing at 95 °C for 5 min, followed by 35 cycles of 1 min at 94 °C for denaturation, 1 min at 52 °C, for annealing and 1 min at 72 °C for elongation, and final extension at 72 °C for 7 min. The resultant PCRs were electrophoresed with the negative control (sterile water instead of DNA) and positive controls (DNA from previous sequencing) on a 1.5% agarose gel and visualized by *Gelview* staining. All *mtCOI* PCR products (~820 bp) were directly sequenced bi-directionally by ABI 3730 DNA analyzer at Sangon Biotech in Shanghai. The sequences were assembled using the CAP3 sequence assembly program (http://doua.prabi.fr/software/cap3). Confirmations of the *B*. *tabaci* species were obtained using the 773 bp assembled sequence analysis.

Each of the 396 *mtCOI* sequences obtained from *B*. *tabaci* Q were checked for gaps, indels, numts, and pseudogenes by alignment using Clustal W in MEGA 5.05^31, 44, 45^. Two different length sequences consisting of 657 bp and 773 bp were analyzed according to the technique devised by De Barro and Ahmed^3^. Sequence similarity of the detected genotypes was analyzed using BLAST on nucleotide sequences deposited in the GenBank database. When at least one mutational site occurred between two *B*. *tabaci* Q sequences, we defined them as two haplotypes. The haplotypes of the 396 *B*. *tabaci* Q were determined by *mtCOI* sequences using DnaSP version 4.10.0.3^46^ and subsequently designated as Q1H1 to Q1H5. The haplotype identifier refers to haplotypes belonging to *B*. *tabaci* Q where there were at least two identical sequences.

### Haplotype diversity analysis of *Bemisia tabaci* Q

The haplotype network of *mtCOI* genes was inferred using the median-joining algorithm based 657 bp and 773 bp, respectively^45, 47^. All calculations were conducted using the software program Network v.4.6.1.0 (Fluxus Technology Ltd., England). The geographical regions were demarcated according to the standard as the under website. (https://figshare.com/articles/_Geographic_regions_of_China_Northeast_China_NE_North_China_NC_Northwest_China_NW_East_China_EC_Central_China_CC_South_China_SC_and_Southwest_China_SW_/1560916).

The genetic diversity indices of each collection which were analyzed based on *mtCOI* using DnaSP v.5.032 included the number of polymorphic (segregating) sites (*S*)^46^, the total number of mutations (*η*)^48^, the average number of nucleotide differences (*K*)^49^, the number of haplotypes (*H*), the haplotype diversity (*Hd*)^50^, the nucleotide diversity (π)^50^, the nucleotide diversity with Jukes and Cantor correction (π (JC))^51^, and the number of net nucleotide substitutions per site between collections with Jukes and Cantor correction, Da (JC)^50^. Tajima’s D (*D*)^49^ and Fu ’s F test^52^ were conducted to detect deviation from neutrality.

## Electronic supplementary material

Dataset 1

## References

[1] Wan FH, Yang NW (2016). Invasion and management of agricultural alien insects in China. Ann. Rev. Entomol..

[2] Miura O (2007). Molecular genetic approaches to elucidate the ecological and evolutionary issues associated with biological invasions. Ecol. Res..

[3] De Barro PJ, Ahmed MZ (2011). Genetic networking of the *Bemisia tabaci* cryptic species complex reveals pattern of biological invasions. PLoS ONE.

[4] Karsten M, van Vuuren BJ, Barnaud A, Terblanche JS (2013). Population genetics of *Ceratitis capitata* in South Africa: implications for dispersal and pest management. PLoS ONE..

[5] Saltonstall K (2002). Cryptic invasion by a non-native genotype of the common reed, *Phragmites australis*, into North America. P. Natl. Acad. Sci. USA.

[6] Zhang RM (2014). Two putative bridgehead populations of *Aphelinus mali* (Hymenoptera: Aphelinidae) introduction in China as revealed by mitochondrial DNA maker. FLa Entomol..

[7] Andraca-Gómez G (2015). A potential invasion route of *Cactoblastis cactorum* within the Caribbean region matches historical hurricane trajectories. Biol Invasions..

[8] Kelly D, Muirhead J, Heath D, Macisaac H (2006). Contrasting patterns in genetic diversity following multiple invasions of fresh and brackish waters. Mol Ecol..

[9] Lee P, Patel R, Conlan R, Wainwright S, Hipkin C (2004). Comparison of genetic diversities in native and alien populations of Hoary Mustard (*Hirschfeldia incana* [L.] lagreze-Fossat). Intl J Plant Sci.

[10] Tsutsui ND, Suarez AV, Holway DA, Case TJ (2000). Reduced genetic variation and the success of an invasive species. P. Natl. Acad. Sci. USA.

[11] Jones DR (2003). Plant viruses transmitted by whiteflies. Eur. J. Plant Pathol..

[12] De Barro PJ, Liu SS, Boykin LM, Dinsdale AB (2011). Species status of *Bemisia tabaci*. Ann. Rev. Entomol..

[13] Wan FH (2009). Invasive mechanism and management strategy of *Bemisia tabaci* (Gennadius) biotype B: progress report of 973 Program on invasive alien species in China. Sci. Chin. Ser. C Life Sci..

[14] Gnankine O (2013). Distribution of *Bemisia tabaci* (Homoptera: Aleyrodidae) biotypes and their associated symbiotic bacteria on host plants in West Africa. Insect Conserv. Diver..

[15] Chu D, Wan FH, Zhang YJ, Brown JK (2010). Change in the biotype composition of *Bemisia tabaci* in Shandong Province of China from 2005 to 2008. Environ. Entomol..

[16] Chu D, Zhang YJ, Wan FH (2010). Cryptic invasion of the exotic *Bemisia tabaci* biotype Q occurred widespread in Shandong Province of China. FLa. Entomol..

[17] Hu J (2011). An extensive field survey combined with phylogenetic analysis reveals rapid and widespread invasion of two alien whiteflies in China. PLoS ONE.

[18] Pan HP (2011). Further spread of and domination by *Bemisia tabaci* biotype Q on field crops in China. J. Econ. Entomol..

[19] Teng X, Wan FH, Chu D (2010). *Bemisia tabaci* biotype Q dominates other biotypes across China. FLa. Entomol..

[20] Pan HP (2015). Insecticides promote viral outbreaks in China by altering herbivore competition. Ecol. Appl..

[21] Hebert PDN, Cywinska A, Ball SL, de Waard JR (2003). Biological identifications through DNA barcodes. Pro. R. Soc. B Biol. Sci..

[22] McKenzie CL (2009). Distribution of *Bemisia tabaci* (Hemiptera: Aleyrodidae) biotypes in Florida-investigating the Q invasion. J. Econ. Entomol..

[23] McKenzie CL (2012). Distribution of *Bemisia tabaci* (Hemiptera: Aleyrodidae) biotypes in North America after the Q invasion. J. Econ. Entomol..

[24] Chu D (2008). Genetic differentiation of *Bemisia tabaci* (Gennadius) (Hemiptera: Aleyrodidae) biotype Q based on mitochondrial DNA markers. Insect Science..

[25] Chu D (2011). Investigation of the genetic diversity of an invasive whitefly (*Bemisia tabaci*) in China using both mitochondrial and nuclear DNA markers. Bull. Entomol. Res..

[26] Li HR (2016). Species identification of indigenous *Bemisia tabaci* agricultural areas in China. J. Plant Protect.

[27] Tsagkarakou A (2007). Biotype status and genetic polymorphism of the whitefly *Bemisia tabaci* (Hemiptera: Aleyrodidae) in Greece: mitochondrial DNA and microsatellites. Bull. Entomol. Res..

[28] Chu D (2008). Genetic differentiation of *Bemisia tabaci* (Gennadius) (Hemiptera: Aleyrodidae) biotype Q based on mitochondrial DNA markers. Insect Sci..

[29] Chu D (2013). Spatial genetic heterogeneity in populations of a newly invasive whitefly in China revealed by a nation-wide field survey. PLoS ONE.

[30] Chu D (2014). Evidence for rapid spatiotemporal changes in genetic structure of an alien whitefly during initial invasion. Sci. Rep..

[31] Kelly D, Muirhead J, Heath D, Macisaac H (2006). Contrasting patterns in genetic diversity following multiple invasions of fresh and brackish waters. Mol. Ecol.

[32] Xavier R, Santos AM, Lim FP, Branco M (2009). Invasion or invisibility: using genetic and distributional data to investigate the alien or indigenous status of the Atlantic populations of the peracarid isopod, *Stenosoma nadejda* (Rezig 1989). Mol. Ecol..

[33] Goldstien SJ (2011). Global phylogeography of the widely introduced North West Pacific ascidian *Styela clava*. PLoS ONE.

[34] Wares, J. P., Hughes, A. R. & Grosberg, R. K. Mechanisms that drive evolutionary change: insights from species introductions and invasions. In: Sax, D. F., Stachowicz, J. J., Gaines, S. D. eds Species invasions: Insights into ecology, evolution, and biogeography. Sinauer Associates: Massachusetts. 229–257 (2005).

[35] DeHeer CJ, Vargo EL (2008). Strong mitochondrial DNA similarity but low relatedness at microsatellite loci among families within fused colonies of the termite *Reticulitermes flavipes*. Insect. Soc..

[36] Frankham R (2005). Invasion biology-resolving the genetic paradox in invasive species. Heredity..

[37] Yang X (2013). Two cytochrome P450 genes are involved in imidacloprid resistance in field populations of the whitefly, *Bemisia tabaci*, in China. Pestic. Biochem. Phys..

[38] Wang ZY, Yan HF, Yang YH, Wu YD (2010). Biotype and insecticide resistance status of the whitefly *Bemisia tabaci* from China. Pest Manag. Sci..

[39] Mahadav A, Kontsedalov S, Czosnek H, Ghanim M (2009). Thermotolerance and gene expression following heat stress in the whitefly *Bemisia tabaci* B and Q biotypes. Insect Biochem. Mol. Biol..

[40] Pan HP (2012). Factors affecting population dynamics of maternally transmitted endosymbionts in *Bemisia tabaci*. PLoS ONE.

[41] Fang YW (2014). Competitive ability and fitness differences between two introduced populations of the invasive whitefly *Bemisia tabaci* Q in China. PLoS ONE.

[42] Parrella G (2013). Invasion of the Q2 mitochondrial variant of Mediterranean *Bemisia tabaci* in southern Italy: possible role of bacterial endosymbionts. Pest Manag. Sci..

[43] Frohlich D (1999). A phylogeographic analysis of the *Bemisia tabaci* species complex based on mitochondrial DNA markers. Mol. Ecol..

[44] Tamura K (2011). MEGA5: molecular, evolutionary, genetics, analysis, using maximum, likelihood, evolutionary, distance, and maximum, parsimony, methods. Mol. Biol. Evol..

[45] Thompson JD, Higgins DG, Gibson TJ (1994). CLUSTAL W: improving the sensitivity of progressive multiple sequence alignment through sequence weighting, position-specific gap penalties and weight matrix choice. Nucleic Acids Res..

[46] Rozas J, Sanchez-Delbarrio JC, Peypoch XM, Rozas R (2003). DnaSP, DNA polymorphism analyses by the coalescent and other methods. Bioinformatics.

[47] Bandelt HJ, Forster P, Röhl A (1999). Median-joining networks for inferring intraspecific phylogenies. Mol. Biol. Evol..

[48] Watterson GA (1975). On the number of segregating sites in genetical models without recombination. Int. J. Publ. Opin. Res..

[49] Tajima F (1983). Evolutionary relationship of DNA sequences in finite populations. Genet..

[50] Nei, M. Molecular Evolutionary Genetics. Columbia Univ. Press, New York (1987).

[51] Lynch M, Crease TJ (1990). The analysis of population survey data on DNA sequence variation. Mol. Biol. Evol..

[52] Fu YX (1997). Statistical tests of neutrality of mutations against population growth, hitchhiking and background selection. Genet.

