# Supplementary material for: Population genetics of an alien whitefly in China: implications for its dispersal and invasion success
Source: Sci Rep. 2017 May 22;7:2228. doi: 10.1038/s41598-017-02433-5 (PMC5440374; doi:10.1038/s41598-017-02433-5)
Supplement: Supplementary file 1 — Dataset 1 [file 41598_2017_2433_MOESM1_ESM.doc]

**Title**

Population genetics of an alien whitefly in China: implications for its dispersal and invasion success

**Author list**

Hong-Ran Li^1*^, Hui-Peng Pan^2*^, Yun-Li Tao^1^, You-Jun Zhang^3**^, Dong Chu^1**^

^1^Key Lab of Integrated Crop Pest Management of Shandong Province, College of Agronomy and Plant Protection, Qingdao Agricultural University, Qingdao, 266109, P. R. China

^2^Department of Entomology, South China Agricultural University, Guangzhou 510642, P. R. China

^3^Institute of Vegetables and Flowers, Chinese Academy of Agricultural Sciences, Beijing, 100081, P. R. China

^*^These authors contributed equally to this work.

^**^Correspondence and requests for materials should be addressed to Dong Chu (Email: [chinachudong@qau.edu.cn)](mailto:chinachudong@qau.edu.cn)), You-Jun Zhang (Email: zhangyoujun@caas.cn)


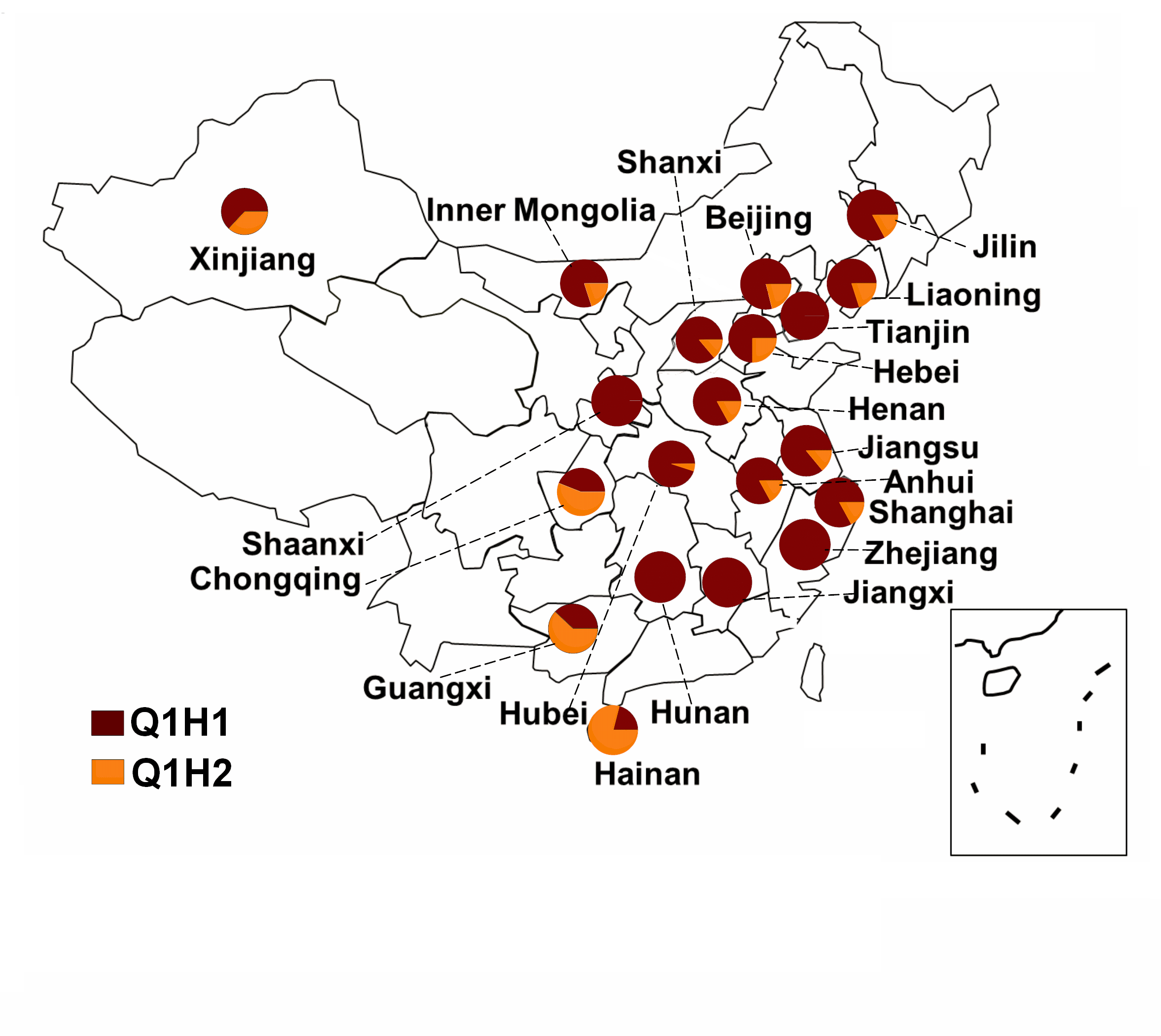


**Supplementary Figure** **S1 | Distribution of different haplotypes among *Bemisia tabaci* Q based 657bp fragment in China in 2011.** Circles denote proportion of Q1H1 (brown) and Q1H2 (yellow) in sampled populations. This China map was created using Microsoft PowerPoint (version 2010) by author Hui-Peng Pan.


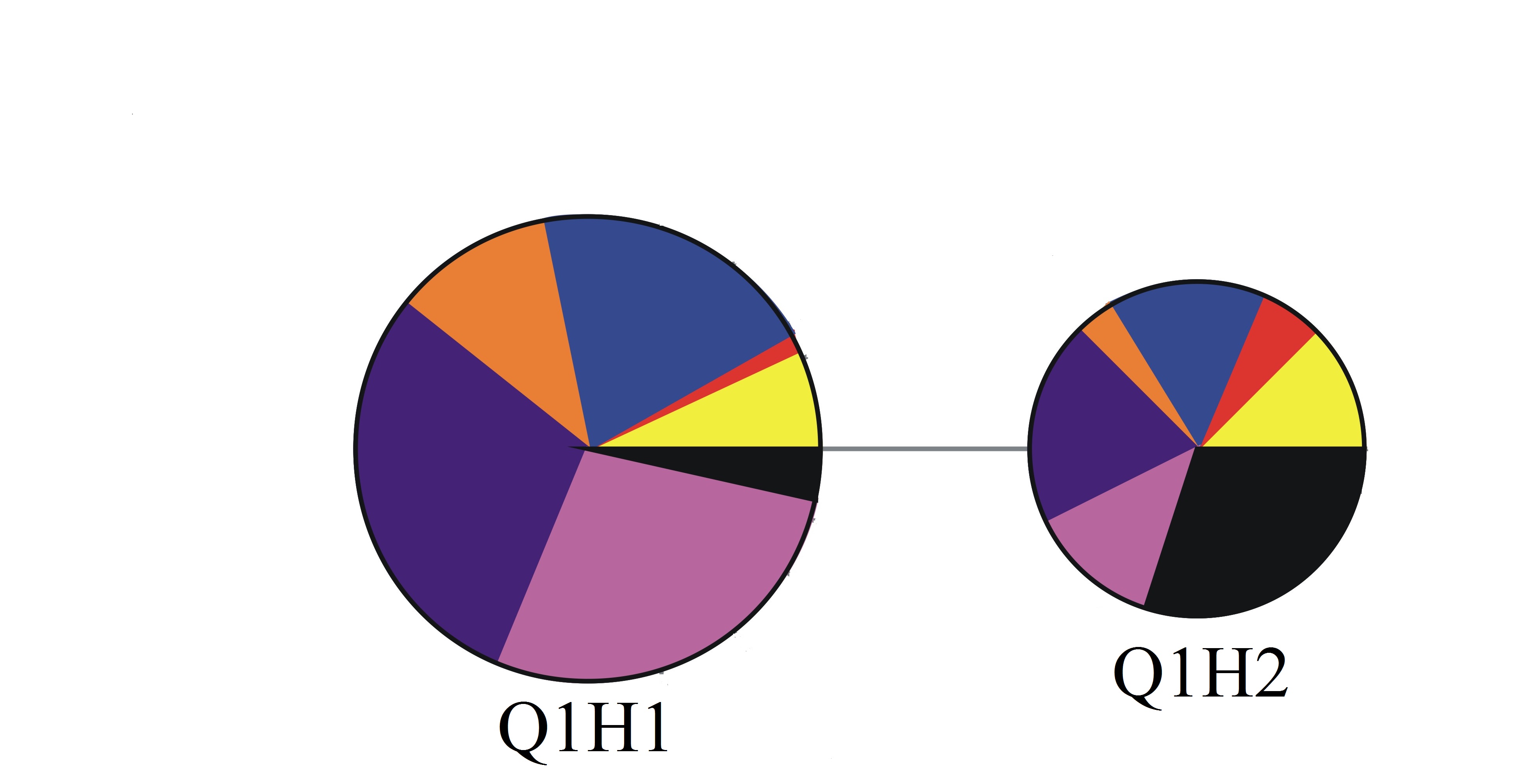


**Supplementary Figure S2 | Network analyses of the haplotype relationships based on 657bp fragment.** Colors within the nodes: red, Southeast China; orange, Central China; yellow, Northwest China; purple, Northeast China; blue, North China; pink, East China; black, South China.


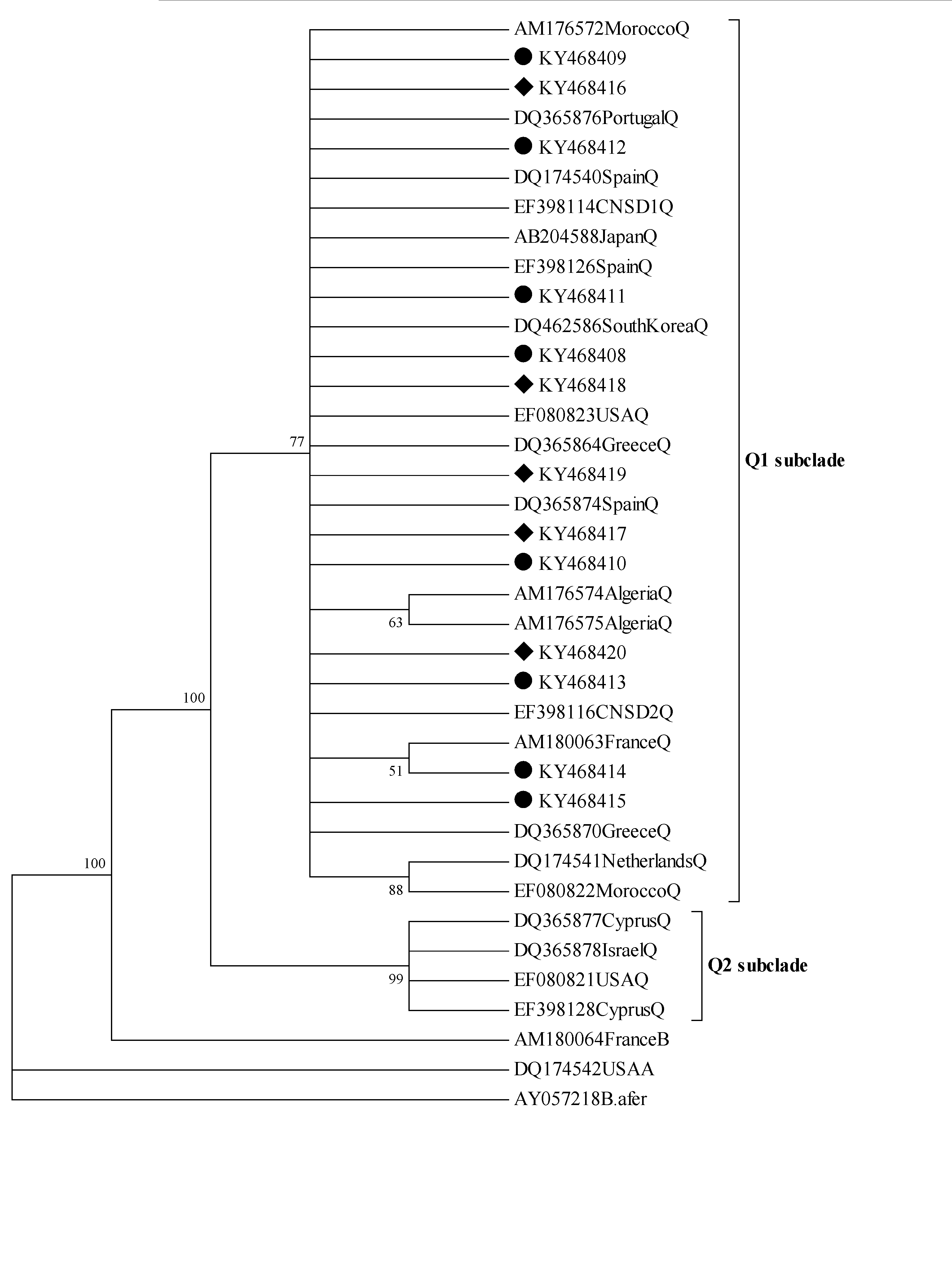


**Supplementary Figure S3** NJ tree for *mtCOI* sequences (~650-bp) of Q1H1-Q1H5 and sequences presented only one time in the present study and *Bemisia tabaci* sequences in Chu *et al*. (2008). The outgroup was *mtCOI* sequence of *Bemisia afer* (AY057218). ⯁: Q1H1-Q1H5. ●: Sequence presented only one time.

**Supplementary Table S1 | Genetic diversity indices of *Bemisia tabaci* Q collections based on 657bp *mtCOI* in China in 2011**

| Population code*  (Number of individuals tested) | *S* | *η* | *H* | *Hd* (SD) | *π* (SD) | *π* (JC) | *K* | *D* (p) | *Fs* (*p*) |
| --- | --- | --- | --- | --- | --- | --- | --- | --- | --- |
| Xinjiang (27) | 1 | 1 | 2 | 0.484 (0.054) | 0.00074(0.00008) | 0.00074 | 0.484 | -1.39947 (ns) | -1.514 (ns) |
| Chongqing (9) | 1 | 1 | 2 | 0.556 (0.090) | 0.00085 (0.00014) | 0.00085 | 0.556 | 1.40117(ns) | 1.015(ns) |
| Inner Mongolia (5) | 1 | 1 | 2 | 0.400 (0.237) | 0.00061 (0.00036) | 0.00061 | 0.400 | -0.81650(ns) | 0.090(ns) |
| Shanxi (15) | 1 | 1 | 2 | 0.248 (0.131) | 0.00038(0.00020) | 0.00038 | 0.248 | -0.39883 (ns) | -0.133 (ns) |
| Hebei (16) | 1 | 1 | 2 | 0.400 (0.114) | 0.00061 (0.00017) | 0.00061 | 0.400 | -0.64998(ns) | -0.872(ns) |
| Beijing (27) | 1 | 1 | 2 | 0.313 (0.096) | 0.00048 (0.00015) | 0.00048 | 0.313 | -0.33572(ns) | -0.753(ns) |
| Tianjin (10) | 0 | 0 | 1 | 0.000 (0.000) | 0.00000 (0.00000) | 0.00000 | 0.000 | - | - |
| Hubei (22) | 1 | 1 | 2 | 0.091 (0.081) | 0.00014 (0.00012) | 0.00014 | 0.091 | -1.16240(ns) | -0.957(ns) |
| Henan (12) | 1 | 1 | 2 | 0.303 (0.147) | 0.00046 (0.00022) | 0.00046 | 0.303 | -0.19492(ns) | -0.297(ns) |
| Jilin (22) | 1 | 1 | 2 | 0.173 (0.101) | 0.00026 (0.00015) | 0.00026 | 0.173 | -0.64112(ns) | -0.176(ns) |
| Liaoning (84) | 1 | 1 | 2 | 0.281 (0.054) | 0.00043 (0.00008) | 0.00043 | 0.281 | -0.48291(ns) | -1.049(ns) |
| Jiangsu (44) | 1 | 1 | 2 | 0.241(0.076) | 0.00037 (0.00012 | 0.00037 | 0.241 | -0.06730(ns) | -0.551(ns) |
| Anhui (12) | 1 | 1 | 2 | 0.303 (0.147) | 0.00046 (0.00022) | 0.00046 | 0.303 | -0.19492(ns) | -0.297(ns) |
| Shanghai (13) | 1 | 1 | 2 | 0.282(0.142) | 0.00043 (0.00022) | 0.00043 | 0.282 | -0.27429(ns) | -0.240(ns) |
| Jiangxi (22) | 0 | 0 | 1 | 0.000 (0.000) | 0.00000 (0.00000) | 0.00000 | 0.000 | - | - |
| Guangxi (21) | 1 | 1 | 2 | 0.495 (0.060) | 0.00075 (0.00009) | 0.00075 | 0.495 | -1.38372(ns) | -1.403(ns) |
| Hainan (14) | 1 | 1 | 2 | 0.363 (0.130) | 0.00055 (0.00020) | 0.00055 | 0.363 | -0.32440(ns) | -0.643(ns) |

**,* the indices for the Shannxi, Hunan and Zhejiang samples were not calculated because the numbers of individuals were below 5; *S*, number of polymorphic (segregating) sites; *η*, total number of mutations; *H*, number of haplotypes; *Hd*, haplotype diversity; *π*, nucleotide diversity; *K*, average number of nucleotide differences; *π*(JC), nucleotide diversity with Jukes and Cantor correction; *D*, Tajima's *D* statistic; *Fs*, Fu 's *F* test statistic; ns, not significant.
